# Effect of Cytochrome C on the Conductance of Asolectin Membranes and the Occurrence of Through Pores at Different pHs

**DOI:** 10.3390/membranes13030268

**Published:** 2023-02-24

**Authors:** Andrey Anosov, Elizaveta Borisova, Elena Smirnova, Eugenia Korepanova, Anatoly Osipov

**Affiliations:** 1 The Department of Medical and Biological Physics, Sechenov First Moscow State Medical University (Sechenov University), 119991 Moscow, Russia; 2 Kotelnikov Institute of Radioengineering and Electronics of RAS, 125009 Moscow, Russia; 3 The Department of General and Medical Biophysics, Pirogov Russian National Research Medical University, 117997 Moscow, Russia

**Keywords:** bilayer lipid membranes, cytochrome c, electrical conductance, lipid pores

## Abstract

The study of the electrical parameters of asolectin bilayer lipid membranes in the presence of cytochrome c (cyt c) at various concentrations showed that an increase in the concentration of cyt c leads to an increase in the membrane conductance and the appearance of through pores. The studied membranes did not contain cardiolipin, which is commonly used in studying the effect of cyt c on membrane permeability. In the presence of cyt c, discrete current fluctuations were recorded. The occurrence of these fluctuations may be associated with the formation of through pores. The diameter of these pores was ~0.8 nm, which is smaller than the size of the cyt c globule (~3 nm). Measurements carried out at pH values from 6.4 to 8.4 showed that the concentration dependence of the membrane conductance increases with increasing pH. To assess the binding of cyt c to the bilayer, we measured the concentration and pH dependences of the difference in surface potentials induced by the unilateral addition of cyt c. The amount of bound cyt c at the same concentrations decreased with increasing pH, which did not correspond to the conductance trend. An analysis of conductance traces leads to the conclusion that an increase in the integral conductance of membranes is associated with an increase in the lifetime of pores. The formation of “long-lived” pores, of which the residence time in the open state is longer than in the closed state, was achieved at various combinations of pHs and cyt c concentrations: the higher the pH, the lower the concentration at which the long-lived pores appeared and, accordingly, a higher conductance was observed. The increase in conductance and the formation of transmembrane pores are not due to the electrostatic interaction between cyt c and the membrane. We hypothesize that an increase in pH leads to a weakening of hydrogen bonds between lipid heads, which allows cyt c molecules to penetrate into the membrane. This disrupts the order of the bilayer and leads to the occurrence of through pores.

## 1. Introduction

The study of the interaction of cyt c with bilayer lipid membranes is of particular interest since similar processes occur in mitochondria when programmed cell death (apoptosis) is triggered. The exact mechanism of the interaction of fatty acids and acid phospholipids with cyt c is unclear. Cyt c interacts with lipid membranes via electrostatic interactions, hydrogen bonds, and hydrophobic effects [1,2,3,4,5,6,7]. Firstly, cyt c can be adsorbed on the membrane surface. This binding is determined by the electrostatic interaction of positively charged cyt c with negative charges on the membrane surface, which is stabilized by hydrogen bonds at low pH values [1]. Secondly, the hydrophobic interaction of fatty acid chains of lipids with the hydrophobic internal structure of cyt c is possible [2,6,8]. Gorbenko et al. [1] showed that at a physiological pH, a shallow cyt c location is assumed, whereas at a pH below 6.0, the protein tends to be inserted into the membrane core. In experiments, these effects manifest themselves when the membrane contains cardiolipin, which is the most likely candidate for the role of an anionic phospholipid that attaches cyt c to the inner mitochondrial membrane [1,8,9,10].

Various physical methods are used to study cyt c–lipid interactions. Tuominen et al. [8] used steady-state absorption and fluorescence spectroscopy to show the presence of the extended lipid anchorage of cyt c to phospholipid membranes. In this mechanism, one of the phospholipid acyl chains protrudes from the membrane and enters the hydrophobic channel in cyt c, whereas the other chains remain in the bilayer. Bernabeu et al. [9] used two-dimensional infrared correlation spectroscopy to study the interaction of cyt c with phospholipids under temperature changes. The presence of a specific interaction between the protein and vesicles containing cardiolipin was revealed. The adsorption of cyt c to anionic lipid bilayers was studied using atomic force microscopy [6]. It was found that cyt c inserts into the bilayer and resides in its hydrophobic core, changing the mechanical properties of the bilayer.

It is well known that the interaction of cyt c with phospholipid membranes containing cardiolipin leads to an increase in membrane permeability [10,11]. Bergstrom et al. [10] used confocal fluorescence microscopy to visualize the leakage of cyt c across the membranes of single giant unilamellar vesicles containing cardiolipin. The authors showed that cyt c leakage occurs only in cardiolipin-containing membranes and attributed the leakage to the opening of lipid pores formed by the cyt c–cardiolipin conjugate. The pore size was estimated from the permeability values by comparing results to those obtained for equinatoxin II, a member of the family of 20 kDa pore-forming toxins from sea anemones. Permeability was also determined by the calculation of diffusive transport. Two obtained approximate pore diameters of ~2 and ~20 nm were reasonably comparable to the diameter of cyt c (~3 nm). The authors suggested that the pore-forming process may be related to the ability of cyt c to induce a negative curvature stress upon binding to cardiolipin-containing membranes.

Kitt et al. [11] used optical-trapping confocal Raman microscopy to investigate the leakage of 3-nitrobenzenesulfonate from large unilamellar vesicles of dipalmitoylphosphatidylcholine and a dipalmitoylphosphatidylcholine–cardiolipin mixture. Cyt c-related leakage was observed only in vesicles containing cardiolipin. These results suggest that cyt c-induced permeability occurs due to the selective interaction of cyt c with cardiolipin. This leads to the unfolding of the protein, whereas the unfolded form interacts with the acyl chains of cardiolipin inside the bilayer, increasing the permeability of the membrane.

In [12,13], it was shown that the cyt c complex with cardiolipin is formed because of the attachment of the cyt c molecule to the membrane surface due to an electrostatic interaction. The subsequent insertion of one or two of the fatty acid chains of cardiolipin into the protein globule, due to hydrophobic interactions, leads to increased cyt c peroxidase activity, which in turn, increases membrane permeability [14]. It was shown in [15,16] that the addition of cyt c and hydrogen peroxide to asolectin membranes containing cardiolipin leads to the formation of pores, the size of which approximately corresponds to the size of a cyt c globule with a diameter of about 3 nm. Note that the peroxidase activity of cyt c leads to damage to the membrane structure (see, for example, [12,13,14,17]). We also note studies that investigated the toxicity of the interaction of cyt c with various nanostructures, such as for example, carbon nanotubes [18] or graphene nanosheets [19].

In [20], an effect of cyt c at different concentrations on the conductance of DIB (droplet interface bilayer) membranes of diphytanoylphosphocholine, cholesterol, and cardiolipin was investigated. Discrete current fluctuations of different amplitudes and durations were registered. The conductance of electrical spikes observed ranged from 1000 to 8000 pS, yielding pore diameter estimates ranging between 1.4 and 4.0 nm. The authors associated the occurrence of current spikes with the electrostatic interaction of cyt c with the membranes.

In [10,15,16,20], pores were registered in membranes containing cardiolipin. The outer mitochondrial membrane, through which cyt c passes during apoptosis, does not contain significant amounts of cardiolipin [21]. The asolectin used in our study is a natural mixture of phospholipids and is similar in composition to the phospholipids of the outer mitochondrial membrane. This makes it possible, to some extent, to simulate the interaction of cyt c with anionic phospholipids of the outer mitochondrial membrane.

In [22], the mitochondrial matrix and cytosolic pH were measured during staurosporine-induced apoptosis. Within an hour, the mitochondrial pH increased from 7.8 to 8.4, and the cytosolic pH decreased from 7.4 to 7.0. These findings indicate that the alteration of the intracellular pH may be an early event that regulates apoptosis.

In this paper, we investigated the influence of pH and cyt c on the electrical characteristics of asolectin bilayer lipid membranes. The acid phospholipids in asolectin can attach cyt c as cardiolipin does. We compared cyt c-induced conductances and membrane surface potential differences at various pH values to find out what are the mechanisms and processes that contribute to increased bilayer lipid membrane conductance.

## 2. Materials and Methods

### 2.1. Lipid, Cytochrome C, and Electrolytes

Asolectin (Avanti Polar Lipids, Alabaster, AL, USA) was used for the formation of planar lipid bilayer membranes (BLMs). Bulk solutions containing 0.1 M KCl and 5 mM Tris-HCl buffer (all reagents were of analytical grade) were used, and pH values were set to 6.4, 7.4, and 8.4. Cyt c from a bovine heart (Merck KGaA, Darmstadt, Germany) water solution of various concentrations (initial concentration being 5 mg/mL) was added to the bulk solutions.

### 2.2. Planar Lipid Bilayer Membranes

The BLMs were formed according to [23] over a 0.5 mm^2^ circular hole in a 1 mm thick wall of a Teflon chamber at a room temperature of 21 ± 1 °C. The wall separated two compartments, each filled with 2.5 mL of the same electrolyte solution. The membrane-forming solution contained 30 mg of lipids dissolved in 1 mL of n-decane. Before each experiment, the vertical wall of the Teflon chamber was covered with a thin layer of dried membrane-forming solution. Once a small droplet (~0.1 µL) of lipid solution was placed below the hole, a bilayer was formed automatically in ~10 min. The formation of the bilayer was followed by capacitance measurements. To estimate the specific capacitance of the membrane, the area of the membrane formed on the hole was determined using a microscope. The specific capacitances of the studied membranes were in the range of 3–4 nF/mm^2^.

Two methods of membrane formation were used in the measurements: a control membrane was formed, and every 10 min, cyt c was added, or a membrane was formed with cyt c previously added in the bulk solution.

The dependences of the conductance of asolectin membranes on cyt c concentration were measured for three pH values. Four control membranes and three membranes for each cyt c concentration were measured at pH 6.4; additionally, 9 and 5 membranes were measured at pH 7.4, and 5 and 3 membranes were measured at pH 8.4.

### 2.3. Electrical Measurements

Ag-AgCl STREF1 electrodes (OHAUS Corporation, Parsippany, NJ, USA) were placed into both compartments of the chamber. The membrane current was measured using a VA-10X amplifier (NPI Electronics GmbH, Tamm, Germany) with a feedback resistance of 5 GΩ and an integration constant of 20 ms. Current fluctuations were recorded with a sampling rate of 1 kHz in a 16-digit ADC (L-Card, Moscow, Russia). All the measurements were carried out at a room temperature of 21 ± 1 °C. 

### 2.4. Measurement of Conductance and Estimation of the Radius of Pores in the Membrane

A constant voltage was applied to the membrane in the voltage clamp mode. The membrane current in the presence of cyt c shows quantized steps, which are usually associated with the formation of through pores. Considering these pores as cylinders and assuming that the electrolyte conductivity in the pore is equal to the conductivity in the solution, we estimated the pore radius *r* by using the well-known formula [24,25]:

(1) $$r=\sqrt{\frac{Gh}{\pi g}},$$

where *h* = 5 nm is the membrane thickness, *G* is the pore conductance, and *g* = 1.04 S/m is the conductivity of a 0.1 M KCl solution at room temperature.

### 2.5. Asymmetric Addition of Cyt C and Measurement of the Difference in Surface Potential

Surface potential is the difference between the membrane surface potential and bulk solution potential. For a symmetrical membrane in the absence of an external voltage, the difference between the surface potentials is zero. When charged molecules are adsorbed on one side of the membrane, the non-zero difference in the surface potential occurs. For homogeneous planar membranes with a homogeneous charge density, the surface potential is well described by the equations of Gouy–Chapman [26]. This model is used in studying the interaction of cyt c with membranes [1]. We used a method which was previously described in [27] for the measurements of the surface potential change with a one-sided cyt c addition to the membranes.

In the absence of cyt c, the surface potential depends on the number of the charged lipids in the membrane. The surface charge density is equal to

(2) $$\sigma_{0}=-\frac{eX}{A},$$

where *A* is the area of a lipid molecule in the bilayer plane, *e* is the electron charge, and *X* is the molar fraction of charged lipids. The negative surface potential $\varphi_{0}$ can be calculated using the Gouy–Chapman equation

(3) $$\sigma_{0}=\sqrt{8\varepsilon \varepsilon_{0}RTC_{KCl}}\sinh\left(\frac{F}{2RT}\varphi_{0}\right),$$

where *F* is the Faraday constant, *R* is the gas constant, *T* is the temperature, ε is the permittivity of water, $\varepsilon_{0}$ is the vacuum permittivity, and $C_{KCl}$ is the concentration of KCl. The one-sided addition of positively charged cyt c leads to the increase in the surface charge by Δσ and the surface potential by Δφ. In this case, the Gouy–Chapman equation is

(4) $$\sigma_{0}+\Delta \sigma =\sqrt{8\varepsilon \varepsilon_{0}RTC_{KCl}}\sinh\left[\frac{F}{2RT}\left(\varphi_{0}+\Delta \varphi \right)\right].$$

The dependence between the surface charge Δσ and the cyt c concentration $C_{cyt}$ in the bulk solution is described by the Boltzmann equation

(5) $$\Delta \sigma =zKC_{cyt}\exp\left[-\frac{F}{RT}\left(z\Delta \varphi +\varphi_{0}\right)\right],$$

where *z* is the number of charges in the cyt c molecule, and *K* is the binding constant. From Equations (3)–(5) we obtained the equation

(6) $$\begin{array}{l} \sqrt{8\varepsilon \varepsilon_{0}RTC_{KCl}}\left\{\sinh\left[\frac{F}{2RT}\left(\varphi_{0}+\Delta \varphi \right)\right]-\sinh\left(\frac{F}{2RT}\varphi_{0}\right)\right\}=\\ zKC_{cyt}\exp\left[-\frac{F}{RT}\left(z\Delta \varphi +\varphi_{0}\right)\right], \end{array}$$

whose numerical solution allows us to find the surface potential difference Δφ.

**Figure 1:**
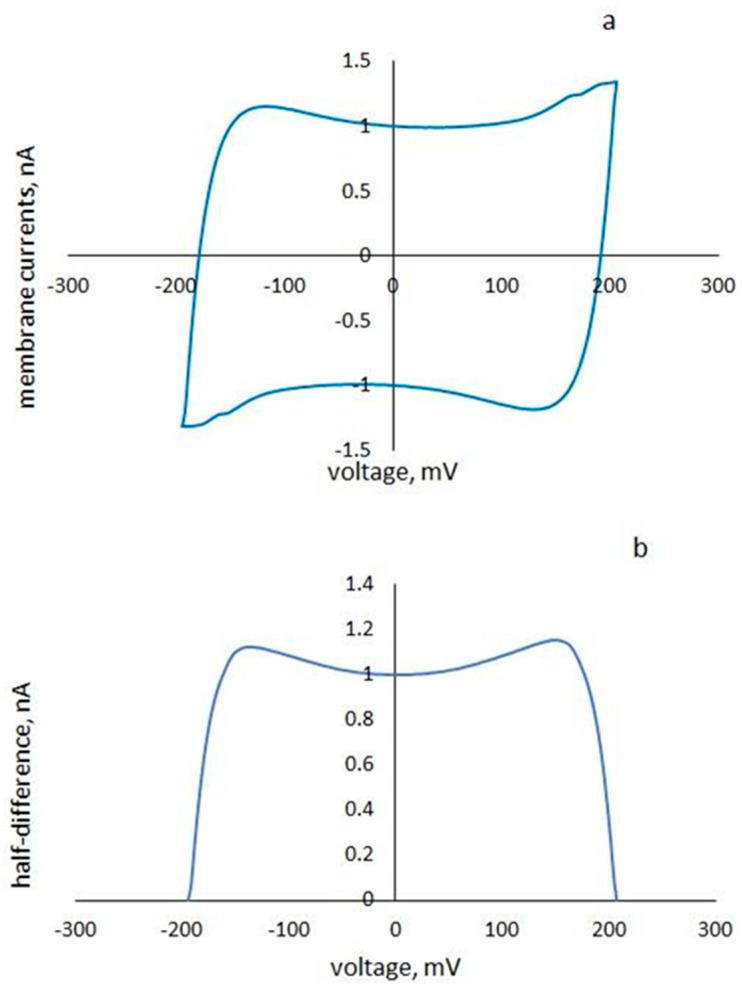
Cyclic current-voltage membrane characteristics (**a**) and the half-difference of the current responses to the upward and downward half-periods of the triangular voltage (**b**).

The measurement method of the surface potential exploits the nonmonotonic voltage dependence of membrane capacitance [28,29,30]. In [29], it was shown that the voltage corresponding to the minimum of the capacitance current could be uniquely calculated using current responses to upward and downward half-periods of the triangular voltage. With an asymmetric addition of cyt c, the resulting difference in the surface potentials can be compensated for by an external command voltage. A triangular alternating command voltage with amplitudes in the range of 100–200 mV and frequencies from 0.5 to 1 Hz was applied to the membrane and cyclic current-voltage characteristics were registered (Figure 1a). The half-difference of the current responses to upward and downward half-periods of the triangular voltage (Figure 1b) is proportional (if transitional processes are excluded) to the membrane capacitance [29]. To assess the difference in surface potentials for each concentration of cyt c and each pH, the voltage corresponding to the minimum half-difference of the current responses was recorded and compared with the corresponding data of the control membranes.

We added the same bulk solution volume to the opposite compartment to eliminate a differential pressure in the chamber caused by the cyt c addition.

## 3. Results

### 3.1. Conductance of Asolectin Membranes

**Figure 2:**
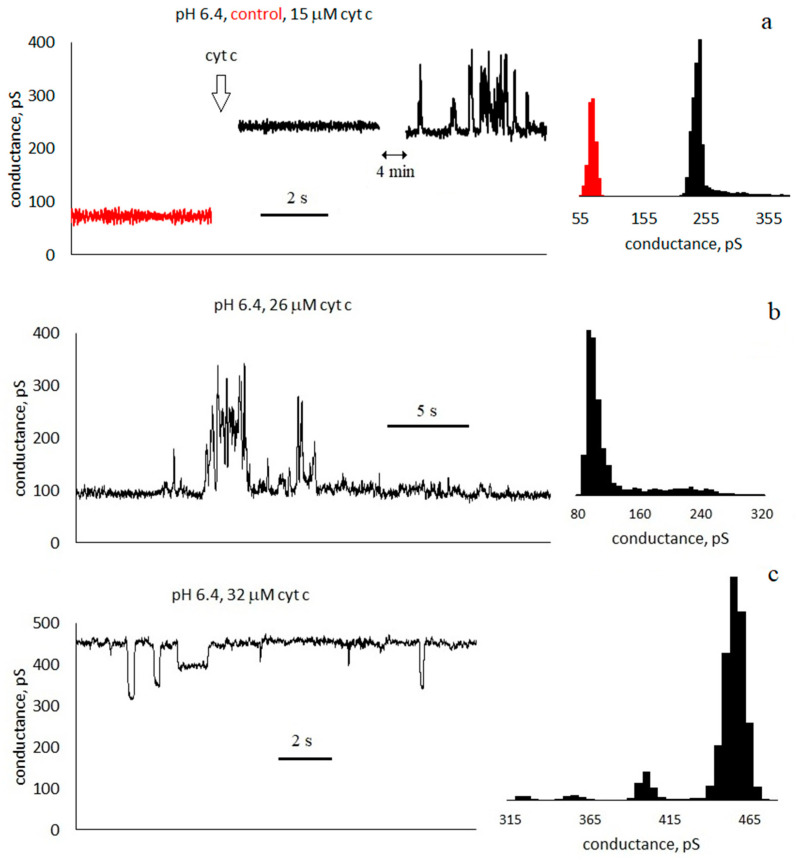
Conductance traces and histograms of asolectin membranes with symmetrical addition of cyt c at pH 6.4: (**a**) control (red) and conductance after addition of 15 μM cyt c, (**b**) 26 μM, and (**c**) 32 μM; 0.1 M KCl, 20 ms averaging.

**Figure 3:**
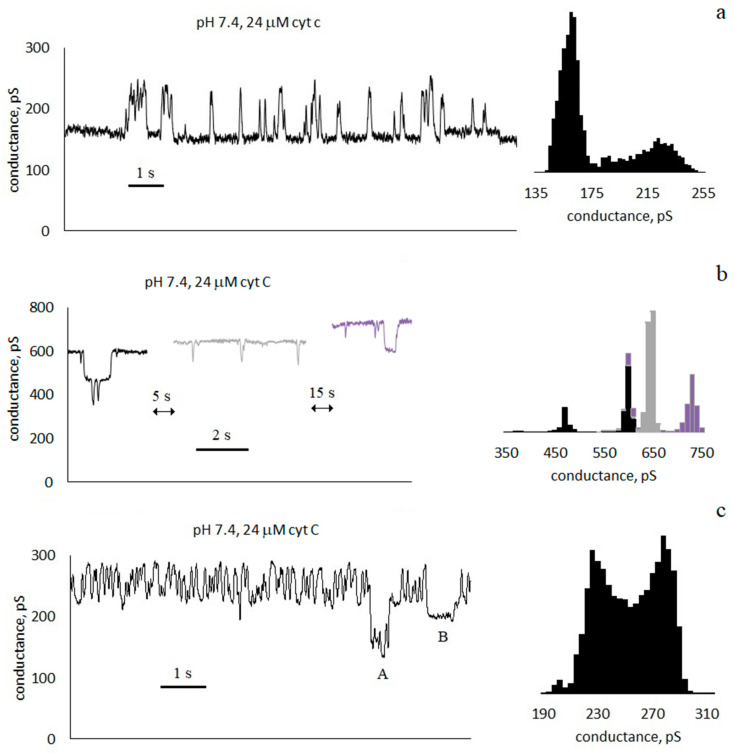
Conductance traces and histograms of asolectin membranes with symmetrical 24 μM addition of cyt c at pH 7.4: (**a**–**c**) are three different membranes; 0.1 M KCl, 20 ms averaging.

**Figure 4:**
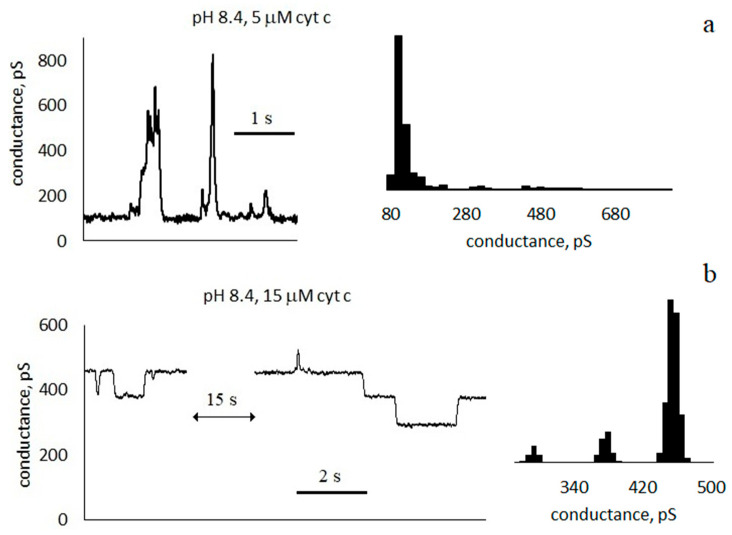
Conductance traces and histograms of asolectin membranes with symmetrical addition of cyt c at pH 8.4: (**a**) 5 μM, and (**b**) 15 μM; 0.1 M KCl, 20 ms averaging.

It was found that membrane conductance increases with the addition of cyt c. Figure 2 shows an example of conductance traces when cyt c is added to a solution surrounding an asolectin membrane at pH 6.4. The control conductance of the membrane was 70 ± 10 pS; after the addition of cyt c, it increased to 240 ± 10 pS. After another 4 min, the basic conductance of the membrane decreased to about 230 ± 10 pS, and pores appeared in the membrane. Conductance traces and histograms are presented in Figure 2, Figure 3 and Figure 4 at pH 6.4, 7.4, and 8.4, respectively.

**Figure 5:**
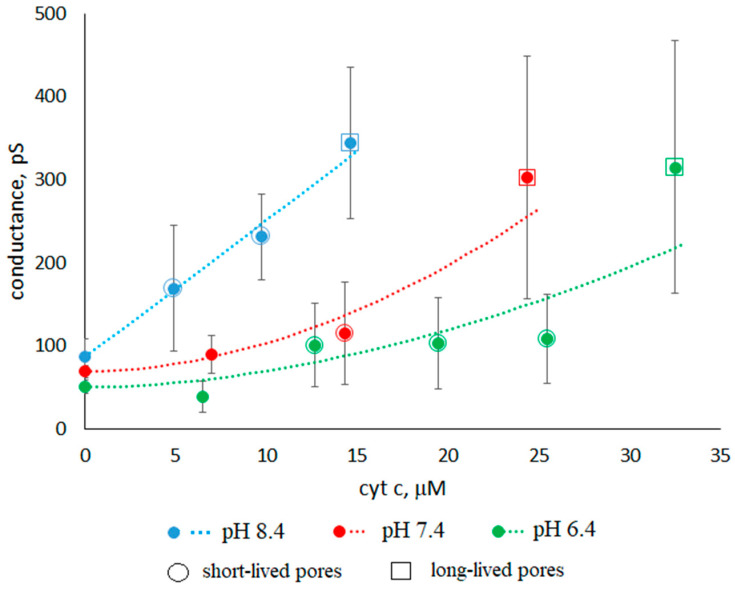
Dependence of the conductance of asolectin membranes on the cyt c concentration *C*. Cyt c additive is symmetrical, 0.1 M KCl. The approximating curves (dotted lines) show an increase in conductance with the addition of cyt c: $0.3\;C^{1.8}$ at pH 6.4, $0.4\;C^{1.9}$ at pH 7.4, and $16\;C^{1.0}$ at pH 8.4. Circles and squares show the appearance of short-lived and long-lived pores, respectively. Standard errors are shown.

Figure 5 shows the dependences of the conductance of asolectin membranes on the concentration of cyt c in the surrounding solution at different pHs. The average conductance was measured at a given concentration one minute after the addition of cyt c. The results obtained show that the membrane conductance increases with an increase in the concentration of cyt c. At the same time, the conductance increases with an increase in pH from 6.4 to 8.4 at the same concentration of added cyt c. Figure 5 shows the standard errors of measurements carried out on different membranes. The variance of conductance from membrane to membrane under the same experimental conditions is significant and increases with an increase in the concentration of cyt c. For example, Figure 3 shows the traces of three membranes measured under the same conditions: pH 7.4 and 24µ M cyt c. The conductances of these three membranes are 170 ± 30, 630 ± 70, and 240 ± 30 pS (SDs are indicated after the ± signs). The average conductance for all three membranes is 350 ± 140 pS (SE is indicated after the ± sign).

We approximated the experimental dependences at different pHs by using the expression:

$$S=S_{0}+aC^{n},$$

where $S_{0}$ is the conductances of membranes at different pHs without cyt c, and *a* and *n* are the approximating coefficients. For linear approximation, we took the logarithm of the conductance increment $S-S_{0}$ (see Supplementary Materials, Figure S1). At pH 6.4, 7.4, and 8.4 we obtained the expressions $S-S_{0}=\left(0.3\pm 0.2\right)\;C^{1.8\pm 0.4}$, $\left(0.4\pm 0.3\right)C^{1.9\pm 0.5}$, and $\left(16\pm 9\right)\;C^{1.0\pm 0.4}$ (see Figure 5, dotted lines), respectively. 

The concentration of cyt c varied from 5 to 33 µM depending on pH. When the conductance reached ~300 pS, the membranes were destroyed. This occurred for the 15 µM cyt c concentration at pH 8.4, for 25 µM at pH 7.4, and for 32 µM at pH 6.4. We believe that the causes of membrane instability are associated with the formation of a large number of defects. This effect is similar to that of SDS at subsolubilizing concentrations on asolectin membranes [31].

### 3.2. Characteristics of Through Pores

The increase in conductance is accompanied by the appearance of discrete current fluctuations, which are usually associated with the appearance of through pores in the membrane (see Figure 2, Figure 3 and Figure 4). The appearance of pores is a random event, and therefore, conductance fluctuations are not recorded in all membranes. At pH 6.4, with a cyt c concentration greater than 12 μM, pores appeared in 71% of membranes. At pH 7.4, with a cyt c concentration greater than 14 μM, pores appeared in 63% of membranes. At pH 8.4, pores appeared in 86% of membranes when cyt c was added. The registered current pulses are characterized by four parameters: shape, amplitude, duration τ, and frequency of occurrence *f*. Rectangular (Figure 2c, Figure 3 and Figure 4b) and non-rectangular (Figure 2b and Figure 4a) pulses were obtained. The rectangular shape of the pulse corresponds to the formation of pores of a constant size. The radius of an equivalent cylindrical pore can be estimated by Formula (1). The observed rectangular pulses correspond to pores with a conductance of 100 ± 40 pS, which corresponds to pore radii in the range of 0.3–0.5 nm. Pulses with a short duration, close to the amplifier time constant of 20 ms, are distorted, have a triangular shape, and have a lower amplitude (Figure 2c, Figure 3 and Figure 4b). Note that the pore sizes are smaller than the size of the cyt c globule.

The temporal characteristics of the observed pulses differ greatly. Conventionally, they can be divided into three groups:

- Pulses with a duration much less than the interpulse interval τ≪1f. For the trace in Figure 3a, the average pulse duration is τ = 100 ± 90 ms, and the frequency is *f* ≈ 2 Hz).
- Pulses with a duration approximately equal to half of the interpulse interval. For the trace in Figure 3c, the average pulse duration is τ = 60 ± 40 ms, and the frequency is *f* ≈ 8 Hz. In this case, in the conductance histogram there are two peaks of approximately the same size. The distributions of the durations of the opened and closed states are presented in the Supplementary Materials (Figure S2).
- Pulses with a duration slightly less than the interpulse interval τ≲1f (Figure 2c, Figure 3b and Figure 4). For the membrane in Figure 3b, the average pulse duration is τ = 1.5 ± 1.9 s, and the frequency is *f* ≈ 0.6 Hz).

We will conditionally call the pores of the first and third groups “short-lived” and “long-lived”, respectively. The concentrations of cyt c and membrane conductances at which short-lived and long-lived pores are observed are shown in Figure 5 by circles and squares, respectively. For example, at a pH of 7.4, a concentration of 14 µM, and a conductivity of 115 pS, a circle is drawn. This means that short-lived pores are registered in the membranes under these conditions. As can be seen from Figure 5, long-lived pores appear at higher cyt c concentrations. At all pH values, the presence of long-lived pores correlates with the high integral conductance of the membrane.

We also note that the appearance of pores is not a stationary process. This can be seen, for example, in Figure 2b: a burst (or two bursts) of conductance with a duration of ~10 s was registered, during which several (more than 10) pores appeared. However, neither before nor after that for 20 s were there any pores.

### 3.3. The Dependence of the Cyt C-Induced Difference in the Surface Potentials on pH

**Figure 6:**
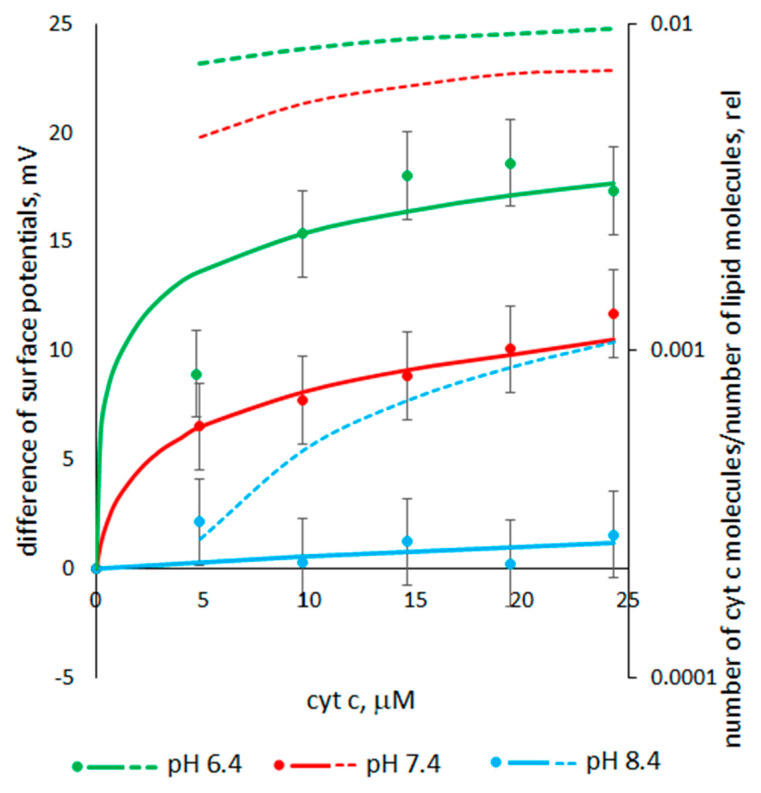
Experimental (markers) and calculated (curves) dependences of surface potential differences, as well as calculated dependences (dotted line) of the ratio of the number of cyt c molecules adsorbed on the membrane to the number of lipid molecules in the membrane, on the cyt c concentration at different pHs: 6.4 (green), 7.4 (red), and 8.4 (blue). Calculated parameters: for pH 6.4, the binding constant *K* = 3000 C·m^−2^·M^−1^, and the number of charges of the cyt c molecule z = +8.5; for pH 7.4, *K* = 110 C·m^−2^·M^−1^, z = +7.25; and for pH 8.4, *K* = 1 C·m^−2^·M^−1^, z = +6. The bulk solution is 0.1 M KCl.

Figure 6 shows the experimental dependences of the difference in the surface potentials on the concentration of cyt c with the one-sided addition to the bulk solution at different pHs. An example of a cyt c-induced shift in the capacitive current minimum is presented in the Supplementary Materials (Figure S3). The results obtained show that the surface potential difference increased with an increasing concentration of cyt c. However, the cyt c-induced potential difference increased more at a lower pH.

Calculating the surface potential in the absence of cyt c, we assumed that for an asolectin membrane at ~ pH 6, the fraction of charged lipids *X* is equal to 23% [30]. In the pH range 6–8.4, according to [32], phosphatidic acid (~3% in asolectin) changes its charge from –1.4 to –2. At pH 8.4, 4% of phosphatidylethanolamine molecules (~20% in asolectin) dissociate and become single-charged anions. In the pH range 6–8.4, phosphatidylinositol (~20% in asolectin) is a single-charged anion and the total charge of phosphatidylcholine (~50% in asolectin) is equal to zero. Thus, at pH 6.4 the percent of charged lipids is equal to *X* = 23.8%, whereas at pH 7.4 it is 24.9%, and at pH 8.4 it is 25.9%. The surface charges $\sigma_{0}$ calculated by Formula (2) are equal to –6.4 (pH 6.4), –6.6 (pH 7.4), and –6.9 (pH 8.4) μC/cm^2^. In this calculation, the area of a lipid molecule in the bilayer plane was considered to be 0.6 nm^2^ [33]. The surface potentials $\varphi_{0}$ calculated by Formula (3) are equal to –66 (pH 6.4), –68 (pH 7.4), and –69 (pH 8.4) mV.

Figure 6 also shows the dependences of the differences in surface potentials on the concentration of added cyt c calculated by Equation (6) at various pH values. The binding constant is used as a fitting parameter. The curves are in good agreement with the experimental data. Figure 6 also shows the dependences of the surface charge Δσ on the concentration of cyt c calculated by Formula (5) at various pH values. The number of cyt c molecules per lipid molecule was determined as ΔσA/ze. The calculation shows that, at pH 6.4 and 7.4, cyt c molecules quite densely cover the membrane surface.

## 4. Discussion

Our measurements of cyt c-induced surface potential differences show that with a decrease in pH, the adsorption of cyt c molecules on the surface of asolectin membranes increases. Meanwhile, this does not lead to an increase in the conductance of the membrane, but on the contrary, the conductance increases with the increase in pH. 

It is known that with an increase in pH from 6.4 to 8.4, the charge of cyt c decreases linearly from +8 to +6.5, or by 19% [34,35]. On the other hand, in this pH range, deprotonation of the polar heads of the acid lipids contained in asolectin occurs, which increases the negative surface charge of the membrane by ~8%. Thus, an increase in pH should lead to the weakening of the electrostatic interaction and to a decrease in the number of cyt c molecules adsorbed on the membrane surface (at a given cyt c concentration), which is observed in the experiment (Figure 6). However, the calculation shows that the binding constant *K* (fitting parameter) increases by about three orders of magnitude with the decrease in pH from 8.4 to 6.4. Such a difference in the values of the binding constants indicates the involvement of other interactions in the binding process. Since the number of protonated molecules sharply increases at a low pH, it can be assumed that the increase in binding between cyt c and lipids is due to an increase in the number of hydrogen bonds between them. This assumption is in line with [1] in which the enhancement of cyt c binding to liposomes containing cardiolipin was obtained at a lower pH. An increase in the number of protein-binding sites on the bilayer surface due to hydrogen bonds with protonated molecules was considered as a possible explanation for this dependence. In turn, such a modification is most likely to involve changes to the acid lipid protonation. Since the acid lipids contained in asolectin are known to exhibit peculiar protonation behavior [32,36], the increase in the amount of partially protonated species is most likely to account for the increased extent of cyt c binding to liposomes at a lower pH.

The data obtained in the experiment (Figure 5) show that the form of the dependence of the membrane conductance on the cyt c concentration strongly depends on pH. At the same concentration of cyt c, the greatest increase in conductance is observed at high pH values. This does not correlate with similar dependences of the surface potential difference and, accordingly, the number of bound cyt c molecules. It is possible that the pH-induced modification of the bilayer surface plays a key role not only in the observed enhancement of cyt c–membrane association at a low pH, but also in the change in permeability. The increase in pH leads to the destruction of hydrogen bonds between lipid heads. It is known that the increase in pH leads to a significant change in the lateral properties of the anion lipid bilayers, where the temperature of the phase transitions from the liquid state to the gel phase decreases, the fluidity rises, and the molecular packing density decreases [32,36,37,38].

Studies performed on monolayers have shown that alteration of the lateral structure of the bilayer at a high pH can lead to increased cyt c-induced changes in the lipid bilayer, facilitating pore formation [39,40]. Cyt c molecules penetrate into the monolayer for low surface pressures and are reversibly squeezed out at higher pressures [41]. A compressibility study showed that the adsorption or intermolecular aggregation of cyt c molecules on the lipid monolayer changes the fluidity of the membrane [7].

The hydrophobic interaction of proteins with membrane lipids is associated with anchoring the penetration of fatty acid chains to the protein thickness. This interaction leads to a change in the conformation of both cyt c and in the properties of the lipid layer. In this case, anchoring can occur in the presence [2] and in the absence [3] of cardiolipin in the membrane. Both electrostatic and hydrophobic interactions lead to the binding of cyt c on the membrane surface, and in our experiment, it is not possible to distinguish one from the other. In any case, the adsorption of cyt c on the surface cannot explain the increase in conductance and the appearance of pores. 

If the protein remains on the membrane surface or anchors, then the cyt c molecule can create a heterogeneous structure in the form of an ordered cluster surrounded by a less ordered structure. The heterogeneity in the membrane can contribute to an increase in the number of defects in it, which can lead to an increase in conductance. The mechanism under consideration is similar to the processes taking place at the phase transitions of lipids. The phase transition from the liquid state to the gel phase is accompanied by the appearance of discrete pores (see, for example, [42]). 

On the other hand, the protein can be immersed into the membrane. This is easier to do at a higher pH when the charge of the cyt c molecule decreases. In [5,6], the immersion of cyt c into membranes of dioleoylphosphatidylglycerol at a low ionic strength was considered. In [6], it was stated that cyt c insertion changes the mechanical properties of the bilayer significantly. Note that anchoring was not considered in [5,6]. The degree of immersion of the cyt c molecule in a bilayer containing cardiolipin was studied in [1]. It has been shown that the estimates of the heme distance from the bilayer center suggest a shallow bilayer location of cyt c at a physiological pH, whereas at a pH below 6.0, the protein tends to insert into the membrane core. These data show that the change in pH affects the immersion of the cyt c molecule in the bilayer, but they are obtained for bilayers containing cardiolipin, and immersion occurs at a pH < 6, whereas our data suggest a possible increase in the cyt c immersion in the bilayer at a pH ~8.4.

It was shown in [10,15,16] that cyt c can form pores in a membrane containing cardiolipin, both in the absence [10] and in the presence of hydrogen peroxide [15,16]. The authors consider the formation of pores to be the result of cyt c peroxidase activity. Note that neither cardiolipin nor hydrogen peroxide were used in our experiments; however, the formation of pores, which is quite intense (see Figure 2, Figure 3 and Figure 4), has been registered. It cannot be excluded that the occurrence of pores is associated with weak cyt c peroxidase activity, but other possible causes of pore formation should not be neglected.

If we assume that cyt c molecules are significantly immersed in the bilayer, then the increase in conductance may not be related to the specifics of cyt c. In this case, the globules disrupt the structure of the bilayer, which can lead to the formation of pores in the membrane. This interaction of cyt c with the bilayer is similar to the interaction of hydrophobic nanoparticles with the membrane. It was shown in [43] that cobalt ferrite nanoparticles form the through pores in asolectin and diphytanoylphosphatidylcholine membranes.

The nonlinearity of the conductance versus concentration at pH 6.4 and 7.4 indicates that the increase in conductance is determined by several concentration-dependent processes. Perhaps these are the ones described above: the adsorption of cyt c on the membrane surface, which increases with the increasing concentration of cyt c (see Figure 6, dotted lines), and the penetration of the protein into the membrane, which is greater, when the concentration of cyt c is greater. Each effect independently increases membrane conductance, but they can act together. In this case, the increase in conductance with an increasing concentration is quadratic, which we can see in Figure 5 at pH 6.4 and 7.4. Comparing the data presented in Figure 5 and Figure 6 at pH 8.4, it can be concluded that the adsorption of cyt c on the membrane surface does not affect the increase in conductance. Consequently, at pH 8.4, only the penetration of the protein into the membrane sets the linear increase in conductance.

Let us consider the pores registered in the experiment. Pores appeared after the cyt c concentration reached 5, 12, and 14 μM at pH 6.4, 7.4, and 8.4, respectively. At higher concentrations the duty cycle τf increases with an increase in the cyt c concentration. This value, τf, determines the average number of pores in the membrane at a given moment. An increase in the lifetime leads to an increase in the integral membrane conductance. Thus, only the duration of the open state of the pore increases significantly with an increase in cyt c concentration. Other parameters of the pore (the size and the frequency of occurrence) are not changed significantly.

Due to the decrease in hydrogen bonds between the polar heads of acid lipids, the packing density of lipid heads decreases with increasing pH. This simplifies the penetration of cyt c molecules into the membrane. The lateral interaction of discrete charges of cyt c can also reduce the packing density. The presence of cyt c globules in the membrane leads to the occurrence of mechanical stresses, increased fluctuations, and the formation of defects, which contributes to the formation of through pores. At high concentrations of cyt c, mechanical stresses in the membrane increase, and the presence of the through pore is energetically more favorable than its absence. This is confirmed by the occurrence of long-lived pores. 

Rectangular current pulses imply the presence of two local minima of energy associated with the open and closed states of the pore. The times of the open τ and closed $\Theta =\frac{1}{f}-\tau$ states of the pore are related to its energy characteristics by these ratios (see, for example, [44]):

(7) $$\tau =\frac{1}{\nu V_{pore}}\exp\left(\frac{E_{max}-E_{open}}{kT}\right),$$

(8) $$\Theta =\frac{1}{\nu V}\exp\left(\frac{E_{max}}{kT}\right),$$

where ν is the attempt rate density of membrane lipids, $V_{pore}$ and V are the membrane volumes whose molecule fluctuations can lead to the closure and opening of the pore, respectively, $E_{max}$ is the value of the energy barrier between the open and closed states of the pore, $E_{open}$ is the energy of the open pore (the energy of the closed pore is $E_{\;close\;}$= 0), *k* is Boltzmann’s constant, and T is the temperature (see Figure S4 in Supplementary Materials). Suppose that in Figure 2c and Figure 3, except for multilevel fluctuations, the same pore closes and opens. In this case, in Equations (7) and (8), the volumes are equal, $V_{pore}=V$, and by dividing the equations, we can find $E_{open}$: $\frac{\tau }{\Theta }=exp\left(-\frac{E_{open}}{kT}\right)$. For the cases shown in Figure 3a and Figure 4a, the difference in energy between the open and closed states of the pore is in units of kT: for short-lived pores, $E_{open}=1.4kT$; for fluctuations in Figure 3c, $E_{open}=0$; and for long-lived pores, $E_{open}=-2.2kT$. The slight differences in the energies of the open and closed states of the pore show that the formation of a pore does not lead to a significant change in the mechanical stresses in the bilayer.

To calculate $E_{max}$, the literature data on the attempt rate density should be used. However, estimates of this value differ by orders of magnitude, and we did not consider it possible to present the results of calculations in the text of this article. They can be found in the Supplementary Materials (Table S1). 

It is worth noting how the pores close by analyzing the intervals A and B in Figure 3c. In both cases, the conductance level decreases. However, in the case of A, fluctuations with a frequency of ~8 Hz do not stop. It follows from this that the pore changing its state with a frequency of 8 Hz has not closed, but the other conductance structure in the membrane has closed. In case B, the fluctuations stop. This means that the observed pore was closed for ~0.5 s. In Figure 2c, for ~4 s, three negative rectangular pulses of different amplitudes are visible. Perhaps it was a closure of three pores of different sizes. However, it is more likely that the same pore changed its size. Moreover, in all three cases, the pore is in a metastable state. Note that a similar result was obtained in [45], where the metastable pores were formed in the diphytanoylphosphatidylcholine membrane, which interacted with cobalt ferrite nanoparticles.

## 5. Conclusions

The cyt c-induced conductance of asolectin membranes depends on its concentration and significantly depends on the pH at a medium range from 6.4 to 8.4. For the same concentration of cyt c, the conductance is greater at a higher pH. Note that in the mitochondria and in the cytosol, the pH values are different and changed in the initial stage of apoptosis. Thus, by changing the pH, it is possible to change the conductance of the membrane with which cyt c interacts.

The unilateral addition of cyt c leads to the appearance of a difference in surface potentials. The measurement of this value at various concentrations and pHs shows an increase in cyt c binding at a low pH. A comparison of the experimental data with the calculation by the Gouy–Chapman formula shows that in order to agree with the experiment at low pH values, the binding constant must be three orders of magnitude higher compared to pH 8.4. This shows that at a low pH, cyt c binding occurs mainly through hydrogen bonding, a hydrophobic interaction, or other mechanisms.

At all three pH values, long-lived pores occur—a state in which a high level of conductance (state of an open pore) is maintained most of the time. The concentration value at which such a state is reached is minimal for a high pH. This suggests that the structure of the lipid bilayer changes in this region due to lateral electrostatic interactions of deprotonated acid lipids and cyt c molecules associated with the bilayer, and due to possible perturbations of the hydrophobic part of the lipid bilayer, which leads to an increase in fluctuations in the bilayer, as well as the appearance of structural defects and mechanical stresses that contribute to the appearance of pores. A possible reason for the increase in conductance at high pH values can also be a decrease in the packing density of lipids in the bilayer and an increase in fluidity due to the breaking of hydrogen bonds between deprotonated acid lipids.

In the presence of cyt c, through pores with a diameter of ~0.8 nm (which is less than the size of the cyt c globule ~3 nm) appear in the membrane. Note that previously, cyt c-induced pores in membranes were registered only in the presence of cardiolipin, which was not used in our study. At low concentrations of cyt c, short-lived pores appear in the membrane, in which the time spent in the open state is less than that spend in the closed state. With an increase in the concentration of cyt c, long-lived pores appear, the lifetime of which is longer in the open state than in the closed one. Thus, the increase in conductance is due, among other things, to an increase in the lifetime of pores, and not to an increase in the frequency of their appearance.

## Data Availability

Not applicable.

## Supplementary Materials

The following supporting information can be downloaded at: https://www.mdpi.com/article/10.3390/membranes13030268/s1, Figure S1: Experimental (markers) and approximating (dotted lines) dependences of conductivity increment on cyt c concentration at different pHs; Figure S2: Distributions of the durations of the open and closed states of pores presented in Figure 2e; Figure S3: The half-difference of membrane currents in control and after one-sided addition of 5 μM cyt c; Figure S4: Energy diagram for the long-lived pore; Table S1: Energy barrier between the open and closed states of a pore. Refs. [46,47] have been cited in supplementary materials.
