# Supplementary material for: Effect of Cytochrome C on the Conductance of Asolectin Membranes and the Occurrence of Through Pores at Different pHs
Source: Membranes (Basel). 2023 Feb 24;13(3):268. doi: 10.3390/membranes13030268 (PMC10053876; doi:10.3390/membranes13030268)
Supplement: Supplementary file 1 [file membranes-13-00268-s001.zip › membranes-2205037-supplementary.pdf]

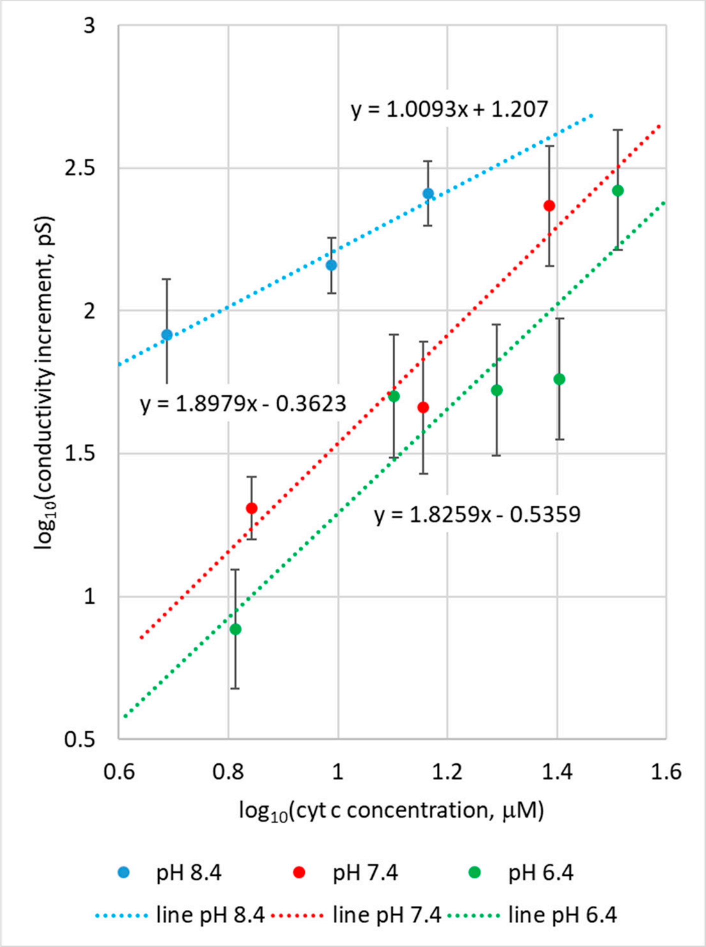

Figure S1. Experimental (markers) and approximating (dotted lines) dependences of conductivity increment on cyt c concentration at different pH.

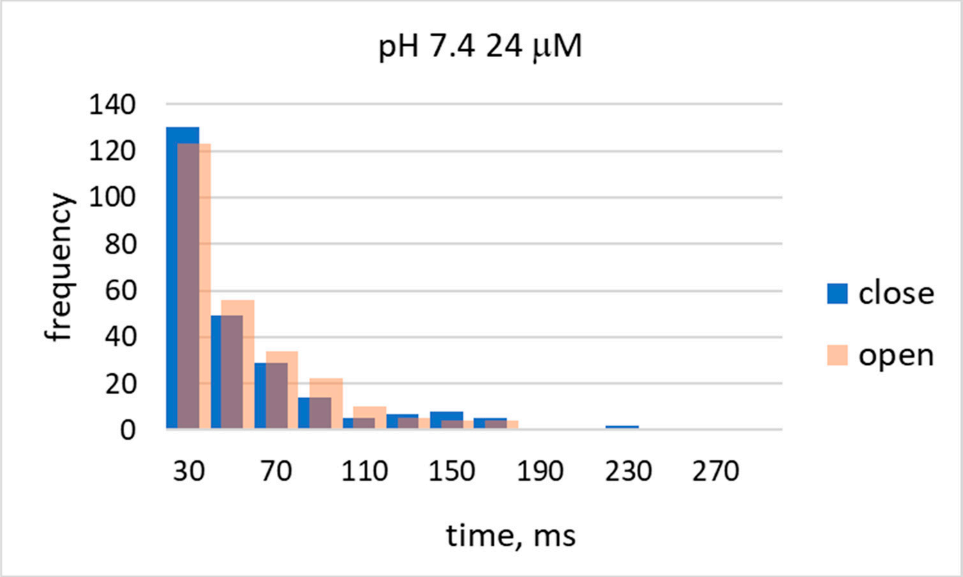

Figure S2. Distributions of the durations of the open and closed states of pore presented in Figure 3c. Voltage +50 mV, 0.1 M KCl, pH=7.4, 24 µM cyt C.

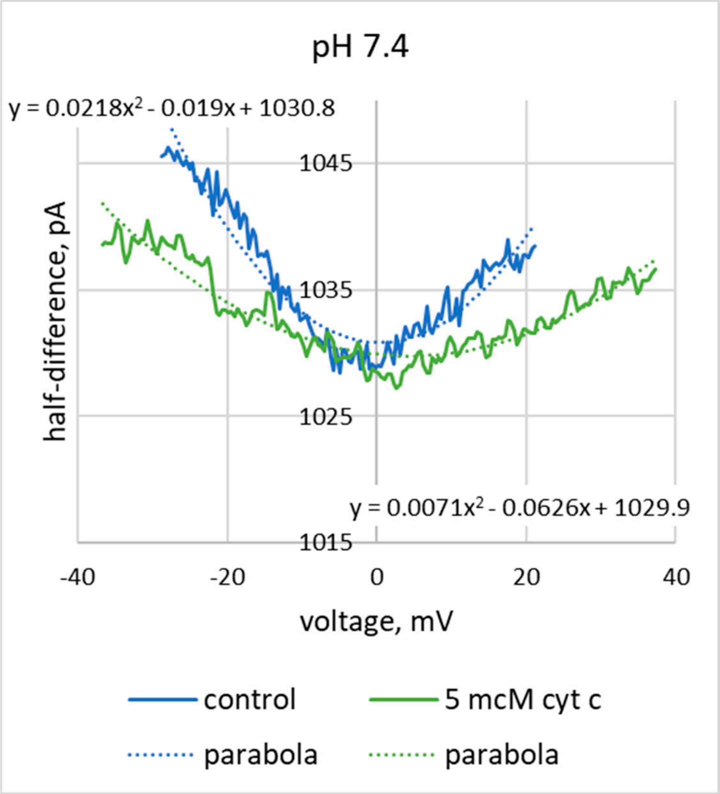

Figure S3. The half-difference of membrane currents in control and after one-side addition of 5 µM cyt c. Parameters of the triangular voltage are 100 mV and 1 Hz. 0.1 M KCl, pH=7.4. The calculated minimum is shifted from 0.4 (control) to 4.4 mV.

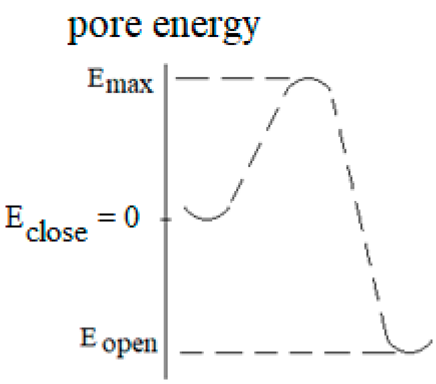

Figure S4. Energy diagram for the long-lived pore $E_{open} < E_{close} = 0$

To estimate E_{max} we can use the literature on value ν. The scatter of ν value is significant – from 5×10^{32} s^{-1} m^{-3} [1] to 2×10^{42} s^{-1} m^{-3} [2]. In [3] we have got for ν 5.6×10^{33} s^{-1} m^{-3}.

According to [2], $V_{pore} = V \approx 2\pi r^2 h$ is the volume of the membrane portion immediately connected with a pore, r = 0.4 nm is the pore radius, h = 5 nm is the membrane thickness.

E_{max} values at different ν are presented in the table:

Table S1. Energy barrier between the open and close states of a pore

ν, s^{-1} m^{-3}	Θ=0.4 s, E_{max}, kT	Θ=0.06 s, E_{max}, kT	reference

5×10^{32}	13.8	11.9	[1]
5.6×10^{33}	16.2	14.3	[3]
2×10^{42}	35.9	34.0	[2]

1. Powell, K. T., Derrick, E. G., & Weaver, J. C. (1986). A quantitative theory of reversible electrical breakdown in bilayer membranes. Bioelectrochemistry and Bioenergetics, 15(2), 243-255. https://doi.org/10.1016/0302-4598(86)80031-9
2. Freeman, S. A., Wang, M. A., & Weaver, J. C. (1994). Theory of electroporation of planar bilayer membranes: predictions of the aqueous area, change in capacitance, and pore-pore separation. Biophysical journal, 67(1), 42-56.
3. Anosov, A. A., Smirnova, E. Y., Ryleeva, E. D., Gligonov, I. A., Korepanova, E. A., & Sharakshane, A. A. (2020). Estimation of the parameters of the Smoluchowski equation describing the occurrence of pores in a bilayer lipid membrane under soft poration. The European Physical Journal E, 43(10), 1-9.
